# Cell type specific adhesion to surfaces functionalised by amine plasma polymers

**DOI:** 10.1038/s41598-020-65889-y

**Published:** 2020-06-09

**Authors:** P. Černochová, L. Blahová, J. Medalová, D. Nečas, M. Michlíček, P. Kaushik, J. Přibyl, J. Bartošíková, A. Manakhov, L. Bačáková, L. Zajíčková

**Affiliations:** 1 Department of Experimental Biology, Faculty of Science, Masaryk University, Kamenice 5, Brno, 625 00 Czech Republic; 2 RG Plasma Technologies, Central European Institute of Technology – CEITEC, Masaryk University, Kamenice 5, Brno, 625 00 Czech Republic; 3 Central European Institute of Technology – CEITEC, Brno University of Technology, Purkyňova 123, Brno, 612 00 Czech Republic; 4 Department of Physical Electronics, Faculty of Science, Masaryk University, Kotlářská 2, Brno, 611 37 Czech Republic; 5 Core Facility Nanobiotechnology, Central European Institute of Technology – CEITEC, Masaryk University, Kamenice 5, Brno, 625 00 Czech Republic; 6 Research Institute of Clinical and Experimental Lymphology– Branch of the ICG SB RAS, 2 Timakova str., 630060 Novosibirsk, Russian Federation; 7 Institute of Physiology of the Czech Academy of Sciences, Vídeňská 1083, Prague, 142 20 Czech Republic

**Keywords:** Biomaterials - cells, Cell adhesion

## Abstract

Our previously-obtained impressive results of highly increased C2C12 mouse myoblast adhesion to amine plasma polymers (PPs) motivated current detailed studies of cell resistance to trypsinization, cell proliferation, motility, and the rate of attachment carried out for fibroblasts (LF), keratinocytes (HaCaT), rat vascular smooth muscle cells (VSMC), and endothelial cells (HUVEC, HSVEC, and CPAE) on three different amine PPs. We demonstrated the striking difference in the resistance to trypsin treatment between endothelial and non-endothelial cells. The increased resistance observed for the non-endothelial cell types was accompanied by an increased rate of cellular attachment, even though spontaneous migration was comparable to the control, i.e., to the standard cultivation surface. As demonstrated on LF fibroblasts, the resistance to trypsin was similar in serum-supplemented and serum-free media, i.e., medium without cell adhesion-mediating proteins. The increased cell adhesion was also confirmed for LF cells by an independent technique, single-cell force spectroscopy. This method, as well as the cell attachment rate, proved the difference among the plasma polymers with different amounts of amine groups, but other investigated techniques could not reveal the differences in the cell behaviour on different amine PPs. Based on all the results, the increased resistance to trypsinization of C2C12, LF, HaCaT, and VSMC cells on amine PPs can be explained most probably by a non-specific cell adhesion such as electrostatic interaction between the cells and amine groups on the material surface, rather than by the receptor-mediated adhesion through serum-derived proteins adsorbed on the PPs.

## Introduction

For many years there is an urgent need in the field of regenerative medicine to develop replacements of non-functional tissues. One way is to carry out the transplantation, but the success of this strategy is complicated by immune reactions to allogeneic or xenogeneic grafts or deficiency of donor tissue. Therefore, many laboratories try with great effort to develop resorbable tissue scaffolds that could support the patient´s cells. The scaffold material should be biocompatible, i.e., non-toxic and non-immunogenic, biodegradable, and at the same time, ecological and easy to manufacture, thus economically viable.

In orthopaedic non‐load bearing devices or dental implants, the preferred materials can be resorbable ceramics chemically similar to the inorganic component of the bone tissue, such as hydroxyapatite^1,2^. However, its biocompatibility should be adjusted to match that of autografts. It improves by increasing the hydrophilicity of the hydroxyapatite surface, especially the inner surface of its pores^3^. Load-bearing implants are preferentially made of metals due to their mechanical properties. The dominant materials became titanium‐based alloys that are generally well tolerated *in vivo*^4,5^. However, these materials fail to encourage osseointegration on the cellular level actively, so the adjustment of their parameters could also be useful^6^.

In soft tissue replacement, a possible solution is to produce a structure made of a biodegradable polymer that mimics extracellular matrix (ECM), which would be peacefully received and gradually degraded when the new tissue has formed^7,8^. The structure is essential because native ECM creates space where the cells are anchored, communicate, proliferate, differentiate, and die. ECM provides mechanical support for cells and also determines the shape of tissue^9^. The synthetic biodegradable polymers like polycaprolactone, poly(l-lactic acid), poly(glycolic acid), poly(lactic-co-glycolic acid) offer simple electrospinning fabrication with controllable nanofibrous morphology, but it is necessary to modify their hydrophobic and inert surfaces^10–12^.

The medical application of materials mentioned above could benefit from plasma processing of material surfaces. Plasma technology for surface modifications has many advantages compared to morphological and chemical treatments, such as low toxicity, short time of production, substrate independence and, if required, a negligible degradation of the original material. Plasma treatment of polymer nanofibers in oxygen or nitrogen-containing discharges is a promising method of how to introduce functional groups that can increase material biocompatibility^13,14^ or can be used to graft additional functionalities^12,15^. However, it leads to functionalization of a near-surface thin layer with rather a short duration due to polymer restructuring^16^. A related problem is the stability of plasma treatment during immersion in aqueous environments^17,18^. On the contrary, the deposition of plasma polymers, i.e., plasma enhanced chemical vapour deposition of thin organic films, can provide a large amount of functional groups on the surface that are more stable^18,19^, because the gradient of their density is not so steep. Plasma polymerization has been reported, e.g., to improve the biocompatibility of vascular stents^20^, favour cell proliferation^21^ and immobilize biomolecules^20,22^.

In this work, we resume on our previous investigation of amine plasma polymers (amine PPs) which surface proved to be efficient for the cell proliferation^23–25^. The study of Manakhov *et al*.^25^ revealed that C2C12 mouse myoblast cells exhibit extreme adhesion to amine PPs-coated surfaces because it was not possible to detach the cells even after a relatively long period of trypsinization. The amine PP coating was also successful for the modification of PCL nanofibrous mats towards their improved biocompatibility and the covalent immobilization of proteins^26,27^. Thus, its function compares to multifunctional polymer films of polydopamine prepared by dip-coating^28^. However, the processing time is much shorter than for the polydopamine films (50 nm after 24 hours) and the technology is compatible with other dry methods for surface patterning.

The phenomena of increased adhesion of cells to amine PPs deserves further investigation, because every cell type requires an appropriate extent of adherence and spreading to survive and proliferate^29^. Here reported results show for the first time that the significantly increased resistance to trypsin can be observed for many different cell types (except endothelial cells) cultivated on amine PP surfaces. The question arises whether the increased resistance of cells to trypsin treatment is associated with increased cell adhesion, and how this affects the cytokinetic parameters. In our work, we applied several different methods and used many different cell types to obtain a more comprehensive picture of the interaction of cells with multifunctional surfaces such as amine PPs. Among them, we investigated the force of cell adhesion and the total work to detach the cell with the single-cell force spectroscopy, which is still a unique and seldom applied method. The method was able to distinguish fine cell-adhesion differences among different functional surfaces with a bit different chemical compositions.

## Results

**Figure 1:**
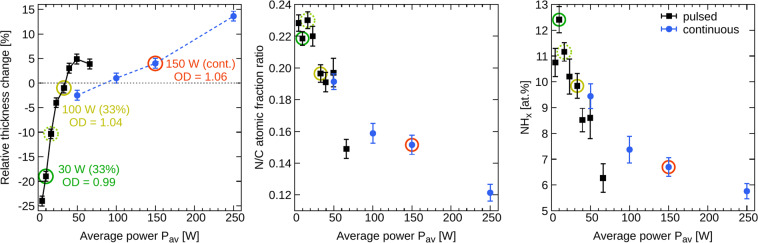
Summary of amine PPs properties depending on the average RF power ${P}_{\text{av}}$. The left graph shows the film stability in water defined as the relative thickness change after 216 hours of immersion in water. The C2C12 cell proliferation assessed by the relative optical density (OD) is shown for the selected ${P}_{\text{av}}$ as the OD numbers. The numbers OD > 1.0 represent the samples that were better than the polystyrene cultivation dish used as a control. The right two graphs provide a summary of the PP compositions determined by XPS (nitrogen-to-carbon ratio and number of amine groups). More details can be found in Manakhov *et al*.^25^. The amine PPs used in the present cell studies are denoted by circles. The error bars represent the standard deviations of mean.

A lower power invested into plasma polymerization process, i.e., lower power of the electrical discharge, leads to a higher retention of the functional group found in the plasma-polymer precursor. Since the discharges cannot be operated at extremely low power, it was proposed to use pulsed (modulated) mode of the discharge to decrease the average invested power ${P}_{\text{av}}$ calculated as the on-time power multiplied by the duty cycle^30,31^. In our previous work, we have studied the plasma polymerization of cyclopropylamine (CPA) in both modes of the radio frequency (RF) discharge, continuous wave (cw) and pulsed^25^. In a simplified picture of the process, the governing parameter related to the film properties (chemical composition and stability in water) was the average RF power, ${P}_{\text{av}}$. The films deposited at low ${P}_{\text{av}}$ were partially soluble but possessed higher nitrogen functionalities (Fig. 1). Increased average power ${P}_{\text{av}}$ decreased the nitrogen-to-carbon (N/C) ratio and NH_x_ atomic percentage as determined with X-ray photoelectron spectroscopy (XPS). It also increased the film crosslinking, and the films became insoluble or exhibited a slight swelling when immersed in water^25^. The latter can be seen from Fig. 1 as an increased relative change of the film thickness after the immersion in water for 216 hours.

It is necessary to clarify that ${P}_{\text{av}}$ is not a unique parameter governing the film chemistry and crosslinking, i.e., solubility. It can be observed from Fig. 1 that the curves belonging to the pulsed and cw modes do not overlap. We have already concluded before^25^ that the best candidates for highly functional yet water-stable films can be prepared in the pulsed mode rather than in the cw mode of RF discharge. Additionally, we wanted to test amine PPs with a high amount of nitrogen, although these films were completely water stable. Therefore, three ${P}_{\text{av}}$ were chosen from the set of pulsed mode experiments, whereas only one ${P}_{\text{av}}$ was selected from the cw mode. The circles in Fig. 1 mark the selected conditions.

The films deposited into Petri dishes were characterized by atomic force microscopy to assess their topography. The deposition of plasma polymers did not alter the roughness character of the unmodified dish (see Figure S1 and Table S1 in Supplementary Information), although it decreased the roughness rms value somewhat (compare 5.7 ± 0.9 nm for uncoated dish with 3.4 ± 0.8, 5.4 ± 1.2 and 4.4 ± 0.8 nm for ${P}_{\text{av}}$ = 10-33 and 150 W, respectively). However, even the unmodified dish exhibited rather small roughness and was quite flat – the ratio between the characteristic height and lateral dimensions of roughness features was smaller than 1:300. Therefore, we do not expect the changes of the surface topography should be considered when discussing differences in the cell-surface interactions.

Water contact angle (WCA) of the uncoated Petri dish was 79°. In the case of amine PPs, it increased with ${P}_{\text{av}}$ from 60° to 76°, which can be explained by a decrease of the N/C ratio. Thus, the PP surfaces were all similar mild hydrophilic and similar to uncoated dish, especially at the highest ${P}_{\text{av}}$.

The previously performed experiments with C2C12 mouse myoblasts indicated that the cells growing on any amine PPs are more resilient to the trypsin treatment than the cells on uncoated dishes^25^. The cells did not detach from amine PPs surfaces even after 30 minutes of trypsinization. Simultaneously, it was shown that the cell proliferation 24 hours after seeding was slightly better on the films prepared at the higher average power. These results are indicated in Fig. 1 as the relative optical density (OD) determined from WST-1 assay (OD > 1 represents the surface that was more suitable for cell adhesion and growth than the uncoated control cultivation dish). Increased cell proliferation on the amine PPs with the lower amount of NH_x_ groups (higher average power) was explained by a better film stability in aqueous media.

**Figure 2:**
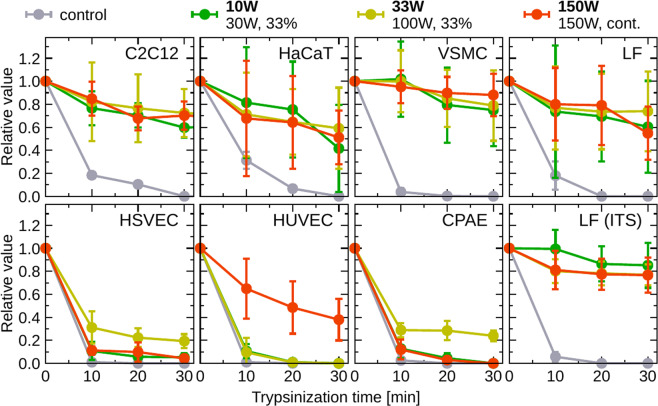
Relative number of cells attached to the surfaces of untreated control Petri dish and three different amine PPs, after different trypsinization periods. Different cell lines, myoblasts (C2C12), fibroblasts (LF), keratinocytes (HaCaT), smooth muscle cells (VSMC), and endothelial cells (HSVEC, HUVEC, CPAE), were cultured using standard conditions described in Methods, Cultivation of Cells. The LF cells were also cultured in serum-free medium and the results are shown in graph labeled LF (ITS). The error bars represent the standard deviations of mean.

The impressive results of highly increased C2C12 cell adhesion to amine PPs motivated current detailed studies. Since mouse myoblasts are not the optimal model for potential use of a newly constructed material in medical applications, similar experiments were performed with human cell lines, fibroblasts (LF) and keratinocytes (HaCaT), that can be used for skin replacements, and rat vascular smooth muscle cells (VSMC) for blood vessels replacements. As shown in Fig. 2, all those cell types behaved similarly as C2C12 when treated by trypsin. 70–90 % of the cells remained attached on the surface even after 30 minutes of trypsinization, and the results did not differ among the tested surfaces in the range of experimental errors. Even if the trypsinization prolonged to 120 minutes, the majority of cells remained attached and alive (data not shown). As demonstrated in Fig. 2, graph labeled LF (ITS), similar results were obtained when the LF fibroblasts were cultured in serum-free medium, i.e., without cell adhesion-mediating protein molecules, such as fibronectin or vitronectin. Within experimental errors, the cell adhesion did not depend on the amount of the amine and amide groups or the film stability, as those changed in the studied surfaces.

Because of the confirmed adhesion of other cell lines to the amine PPs, further experiments were also carried out with primary human endothelial cells (HUVEC, HSVEC) and a bovine endothelial cell line (CPAE) that can be used for engineered vascular grafts embedding cells to generate a living material capable of physiological remodelling^32^. Surprisingly, the endothelial cells behaved differently than the above-discussed cell types. They detached from the amine PP surfaces after the trypsinization in much higher amount and behaved, with some exceptions, similar to the control. For each kind of endothelial cells, there was one type of the amine PP surface, which they favoured. The HSVEC and CPAE cells resisted trypsinization better on the amine PP prepared at ${P}_{\text{av}}$ = 33 W, and the HUVEC cells preferred 150 W amine PP.

**Figure 3:**
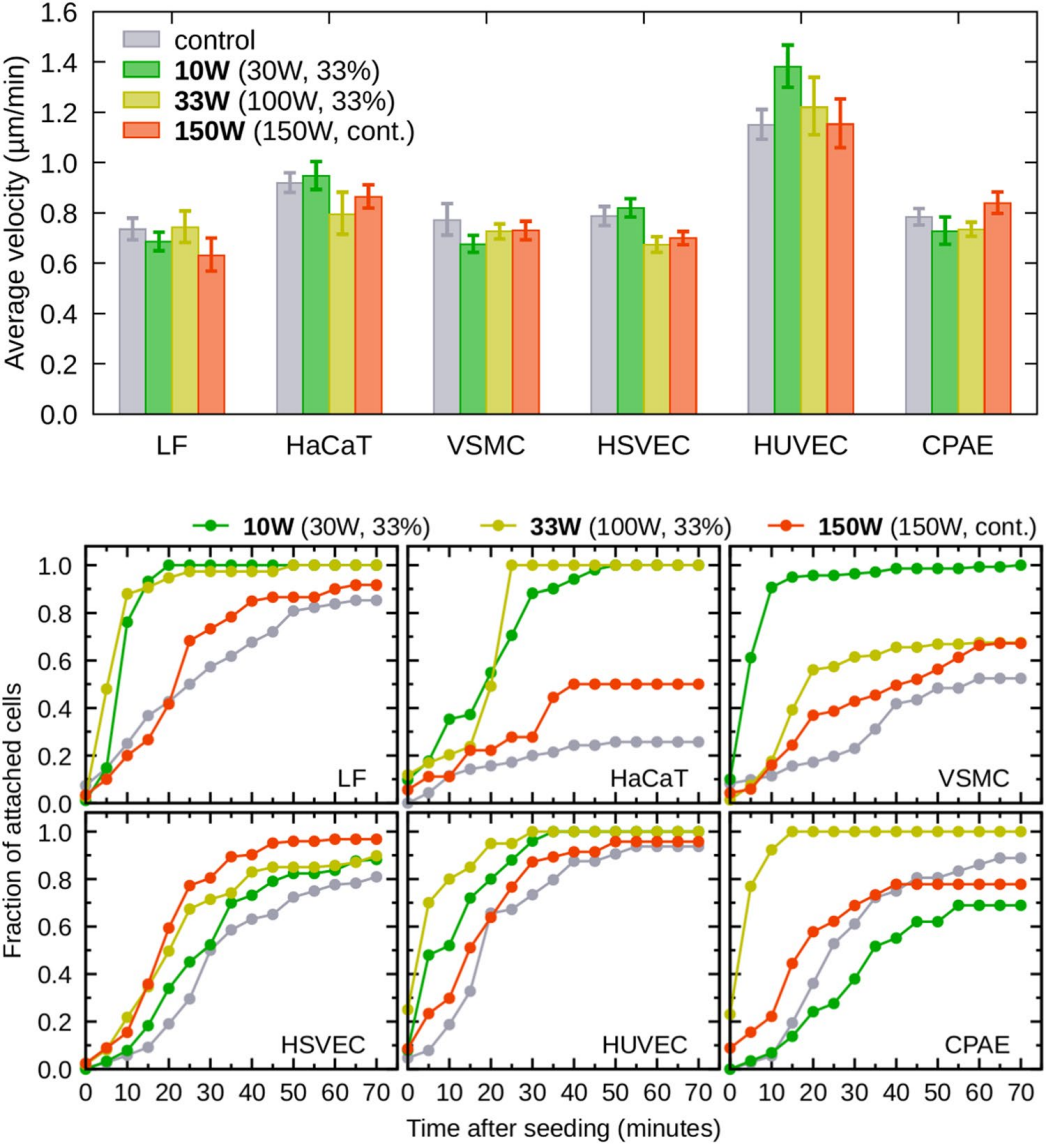
Motility (expressed as an average distance travelled by the cells per minute) and attachment rate of various cell lines. Mostly, the cells attached more quickly to PP-coated dishes (bottom graphs) but their motility (top graphs) did not change from the control (uncoated) Petri dish. The error bars represent the standard deviations of mean.

The above-reported results show for the first time that the significantly increased resistance to trypsin can be observed for many different cell types (except endothelial cells) cultivated on amine plasma polymer surfaces. Therefore, other parameters related to the cell adhesion, such as rate of the cell attachment and cell motility, were evaluated in this work using the time-lapse microscopy of live cells cultivated on uncoated and plasma-coated Petri dishes. The cell motility on amine PPs, evaluated as the average distance that the cells migrated per minute, was similar for any cell type and was not affected by the increased resistance to trypsin (Fig. 3, top). Contrary to the cell motility, the rate of attachment observed during the first 75 minutes after the cell seeding exhibited distinct behaviour for amine PPs (Fig. 3, bottom). The most rapid attachment was observed on the PPs with a relatively high amount of amine groups (${P}_{\text{av}}$ = 10 and 33 W). The most stable layer with a lower amount of amines (150 W) did not affect the attachment rate so dramatically.

Next, we investigated whether PPs can affect cell morphology. The optical cell images were taken after ten hours of cultivation (see Supplementary Information, Fig. S2) and evaluated for the average cell length, width, and aspect ratio (length-to-width ratio). Each cell type has its characteristic shape, which more or less does not depend on the surface (Fig. S3). Therefore, the cell morphology is mostly unaffected by the amine PP films. Yet, the cells tend to be slightly longer on the PPs deposited at ${P}_{\text{av}}=10$ and 33 W than on the control, whereas for ${P}_{\text{av}}=150$ W their length decreases again. However, these differences are small and insignificant.

We aimed to visualize the cell-surface interaction by looking at focal adhesion plaques, which are formed by clustering of cell adhesion receptors, mainly integrins. The extracellular parts of these receptors bind the ECM proteins (e.g., vitronectin, fibronectin), adsorbed on the studied surface from the serum supplement of culture media. The intracellular parts communicate with structural proteins, such as paxillin, talin, or vinculin, that are further associated with the actin cytoskeleton^29^.

**Figure 4:**
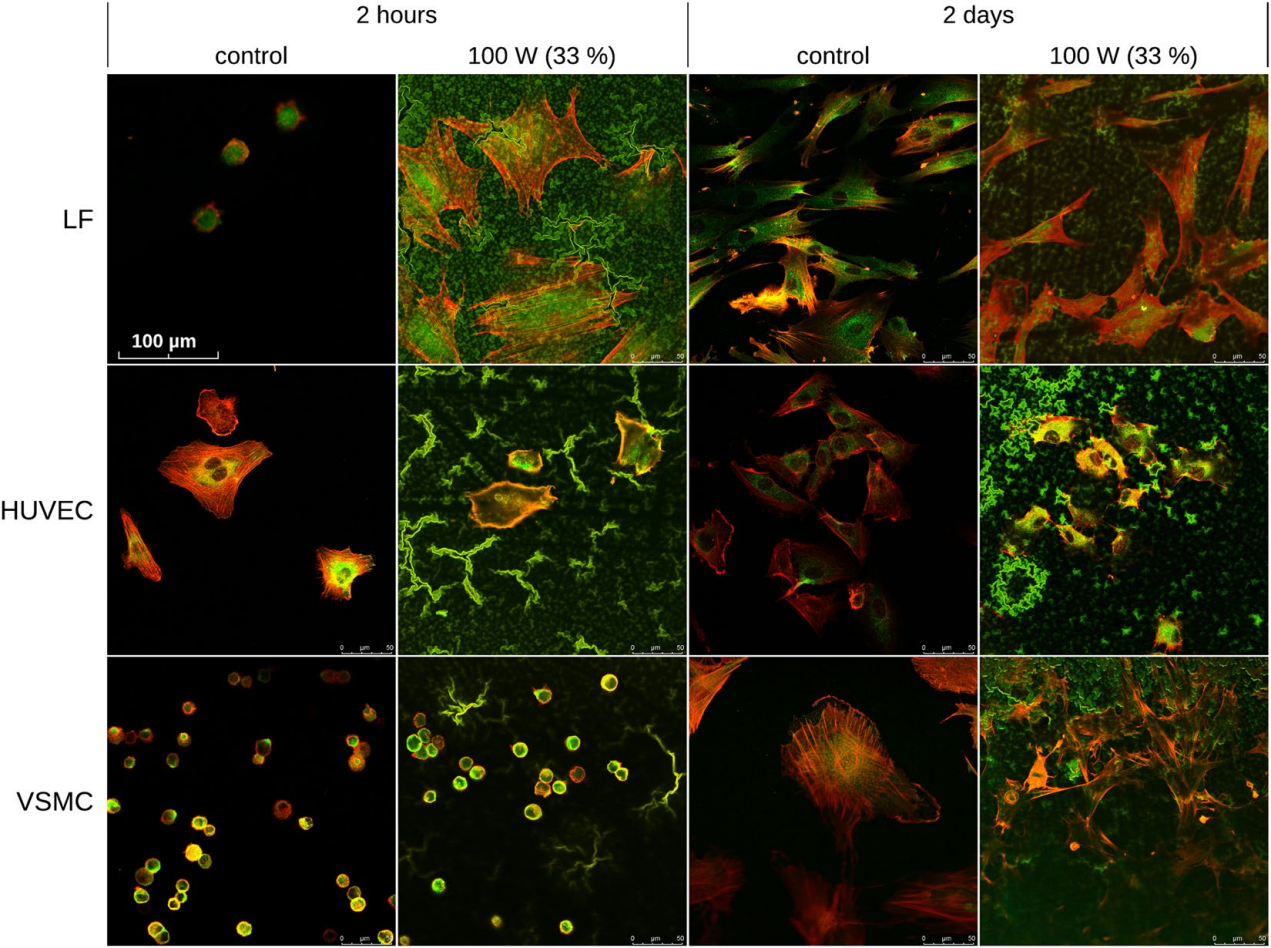
Visualization of cell-surface interaction by immunofluorescence staining of focal adhesion plaques (paxillin, green fluorescence) and by fluorescence staining of actin cytoskeleton by TRITC-conjugated phalloidine (F-actin, red fluorescence). Three cell types (LF, HUVEC and VSMC) were selected as a representative example for the comparison of control (uncoated) and amine PP-coated surfaces. Additional images can be found in Supplementary Information (Fig. S4).

In our study, we visualized (i) the focal adhesion plaques with immunofluorescence staining of paxillin, a protein closely associated with integrin adhesion receptors, and (ii) the actin cytoskeleton with staining of filamentous actin (F-actin) using phalloidin conjugated with a fluorescence marker TRITC. We expected that the visualization of focal adhesion plaques and actin cytoskeleton would provide information on the degree of the specific receptor-mediated adhesion and spreading of cells on the studied surfaces. Thus it would enable to estimate the degree of non-specific direct cell-material interaction that is based, e.g., on electrostatic interactions. In addition, the visualization of focal adhesion plaques and actin cytoskeleton provides information on the size and shape of the cell spreading area. The images for three cell types, i.e., LF, HUVEC, and VSMC, were selected to demonstrate the differences in cell-surface interactions in Fig. 4. All the images are provided in Supplementary Information (Fig. S4).

The interpretation of images was somewhat hampered by the high autofluorescence of the PP surface, especially in the green bandpass. Despite this, we observed that all the cell types were capable of specific receptor-mediated adhesion on the studied surfaces, although its level varied among the cell types and time intervals. In 2-hour-long experiments, the cells displayed two different staining patterns (Figs. 4 and S4 in SI). LF, HaCaT, HUVEC, and CPAE cells were able to spread nicely even in this short time, with the only exception of LF cells growing on control surfaces, which remained mostly roundish. In all these cells, paxillin was visible mainly in the perinuclear region, while F-actin was located at the cell periphery. However, HSVEC and VSMC cells were round in the short experiment on both the PP and control surfaces, and their paxillin expression was peripheral. F-actin was also found on the cell periphery, but it was not assembled into fibers. In 2-day-long experiments, paxillin was visible mainly perinuclearly, and F-actin filaments were located peripherally on all the tested surfaces.

**Figure 5:**
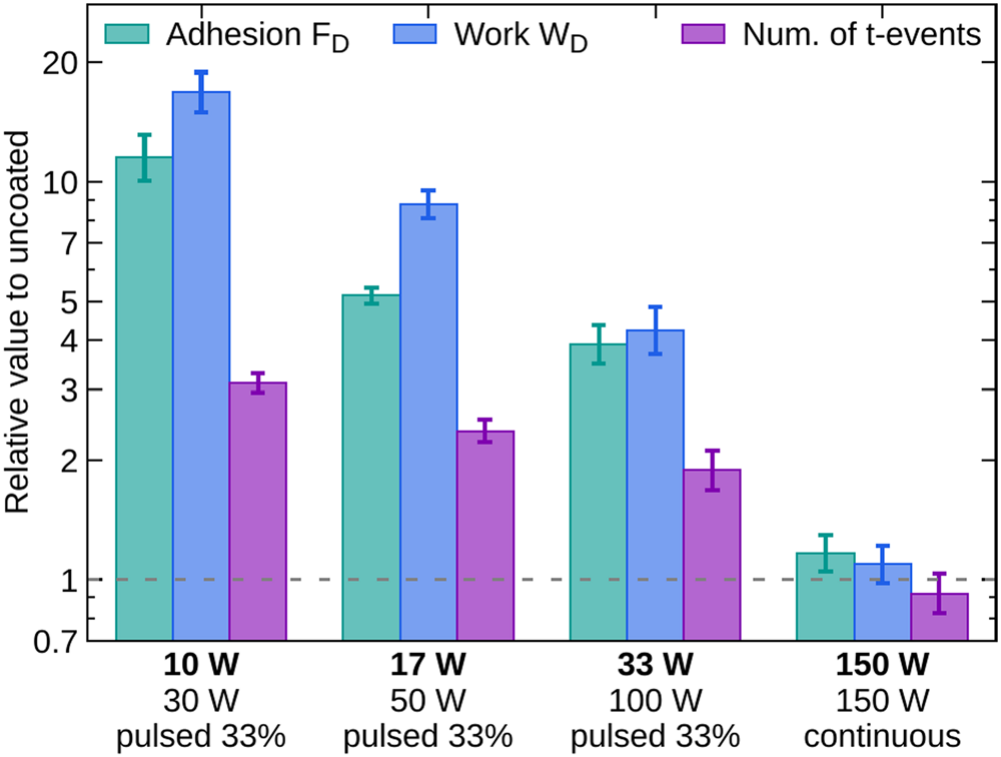
Results of single-cell force spectroscopy for amine PPs deposited at the average power 10, 17, 33, and 150 W. The experiments were carried out with the LF cells. The error bars represent the standard deviations of mean.

The phenomena of increased cell adhesion were also studied by single-cell force microscopy (SCFS) with LF cells. These experiments probed the immediate adhesion because the cells were in contact with the surfaces for only 20 s. The results on the detachment force (maximum adhesion force), F_D_, total work to detach the cell, W_D_, and the number of t-events are summarized in Fig. 5. All three quantities are monotonously increasing with the amount of amine groups, i.e., with the decreasing ${P}_{\text{av}}$ (compare Figs. 5 and 1). The results correlate with the increased rate of attachment on 10 and 33 W PPs. The SCFS results support a natural assumption that if the cell adhesion to amine-containing surfaces is stronger, it should also depend on the amount of amine groups.

**Figure 6:**
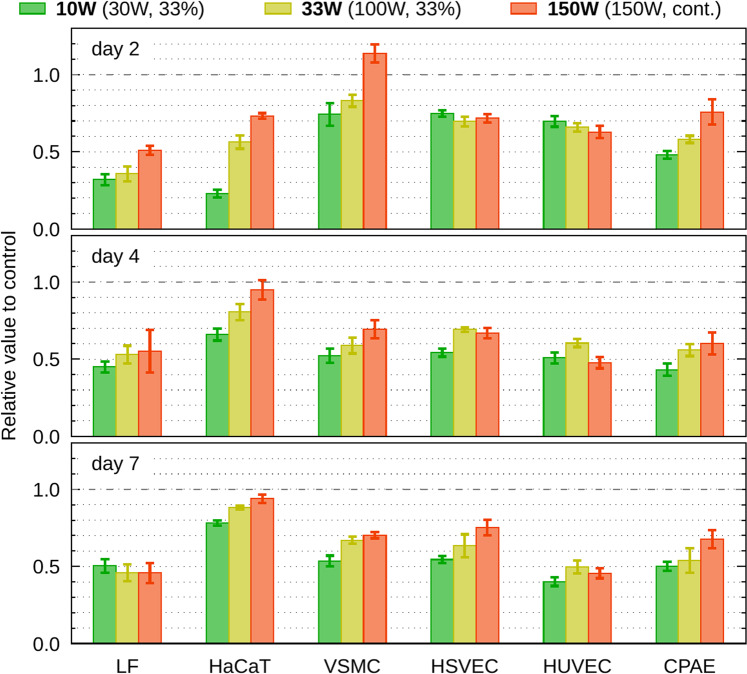
Cell proliferation assessed as the amount of synthetized ATP by cells which grew on PP-coated dishes 2, 4 and 7 days after seeding. The error bars represent the standard deviations of mean.

Since the increased adhesion might negatively affect the replication progress (Uroz *et al*. 2019)^33^, the number of cells on PP-coated dishes was assessed by the total amount of ATP, measured 2, 4, and 7 days after seeding (Fig. 6). The cell growth reduced as compared to the control for all the cell lines and types of the PP film. The PP layer 150 W was the most similar to control surface in majority of cell lines (the HUVEC and HSVEC grew similarly on all the studied surfaces). The PP surface prepared at the ${P}_{\text{av}}$ = 10 W was less suitable for the cell cultivation than the highly crosslinked, more water-stable PP deposited at 150 W. Thus, it is difficult to conclude on a general relation between the increased resistance to trypsinization and cell proliferation. The LF cells exhibited significantly decreased proliferation independently of the PP type, and the cultivation interval, whereas HaCaT and VSMC are almost comparable to the control at the end and beginning of the cultivation, respectively. The resistance to trypsin of the endothelial cell types was not influenced much by the amine PP surfaces (Fig. 2), and despite that, the cells did not proliferate on the PP surfaces better than the other cell types (Fig. 6).

## Discussion

As we reported earlier, the C2C12 cells showed good proliferation activity on amine PP coated dishes in 24 h incubation^25^, but the present work concluded that it was an exclusive case (Fig. 6). The cell proliferation was comparable to the control only in case of HaCaT and VSMC cells, other cell lines were growing slowly on modified dishes. Nevertheless, like for the C2C12 cells, we observed similar strong resistance to trypsin for keratinocytes, fibroblasts, and vascular smooth muscle cells grown on any PP-coated dishes (Fig. 2).

The results of the resistance to trypsinization opened a question if the increased non-endothelial cell adhesion is accompanied with an increased rate of cell attachment and decreased motility. We observed increased attachment rate of the non-endothelial cells to the PPs, but a significantly faster attachment was observed only for the PPs containing a higher number of amine functional groups (Fig. 3, bottom). When testing this result by independent technique (SCFS) on LF cells, we gained the same conclusion – the highest adhesion occurred on layers with a high amount of amines (Fig. 5). We can thus sum up that the short time adhesion is directly proportional to the number of amine groups, but we did not observe the same dependence during long time incubation as the trypsin resistance was comparably strong for all the tested PPs. We can only speculate whether the variance among the PPs is evidenced just in short time incubations because the effect of amine groups is during prolonged incubation replaced or overridden by some other mechanism (see below), or the sensitivity of trypsin resistance measurement is so low, that it wipes off possible differences between different amine PPs. Anyway, when we compare all the cell lines and their parameters, we can see that the PP at 150 W, which has the lowest amount of amine groups, resembles the control dish the most.

Endothelial cells naturally need to resist the stress of blood flow, thus we tested three endothelial cell types (two types of primary cells and one cell line) on the amine PPs for potential future use as active vascular replacements, as endothelialization of the vascular prostheses is considered the best prevention of thrombotic occlusion and restenosis of the graft^34^. To our surprise, the trypsin resilience was much lower in endothelial cells than in the cell types mentioned above, and it was lineage-dependent. Although the endothelial cells on PPs do not resist the trypsin treatment so efficiently and do not migrate slower, their rate of attachment also increases as in the non-endothelial cells. This is also accompanied by decreased proliferation. We can thus conclude that amine PPs increase the adhesion parameters of all the tested cell types. The variability of trypsin resistance of endothelial cells makes them the optimal model for analysing the molecular events behind the trypsin resistance phenomenon. Importantly, all the used cell types are the good physiological models as they are not derived from tumours, neither are artificially immortalized.

The obtained results demonstrated in the previous section provoke many questions: What is the molecular basis of amine groups associated with trypsin resistance? Why do endothelial cells resist less? Why is not HaCaT cell proliferation slowed down even though they can resist trypsin treatment? In the current phase of our research, we cannot put clear evidence to answer them. However, we can offer some very plausible explanations. The cell adhesion parameters increased because of the amine plasma coating of surfaces, which reduced the hydrophobicity of surfaces. It has been proved in many studies before^11,13,35^, but none of them has yet explained the differences between the adhesiveness of various cell types. Many of these studies have reported that plasma-treated polymers have a better influence on the behaviour of cells than untreated polymers, but in our case, human fibroblasts, keratinocytes, and smooth muscle cells showed reduced proliferation to the control dish. Nevertheless, it needs to be stressed out we compared the amine plasma polymer surface with the surface of the cultivation Petri dish, which is optimized (and also plasma treated) for the best cell cultivation. Thus the proliferation decreased to half of the control can still be viewed as very good biocompatibility.

The decreased proliferation could be caused by the increased adhesion strength, manifested by the resistance of the cells to detachment by trypsin. Similar resistance to trypsin treatment is reported in the study of Denstman *et al*.^36^, where the authors explain their results as a change of topology of the attachment-associated membrane proteins leading to the inaccessibility of the cleavage sites of trypsin. A second explanation could be the change of conformation or chemical properties of the cleavage sites. In our case, the amine groups from the surface may change cleavage sites for trypsin as well by oxidizing carboxyl sides of lysine or arginine^36,37^. Thus our inability to detach cells by trypsin could be caused by inefficient cleavage. However, the results on endothelial cells disprove this explanation as they are usually prone to trypsin treatment too.

Appropriate adhesion strength is necessary for starting the cell proliferation. Without sufficient attachment to the support for growth, the anchorage-dependent cells undergo anoikis, which is a specific type of apoptosis caused by adhesion deprivation. However, a very high adhesion strength, which can be due to a large cell spreading area, and numerous and large focal adhesion plaques associated with the well-developed actin cytoskeleton, can hamper the cell proliferation, because it is challenging to disintegrate these complicated structures before the mitosis. Thus, firmly adhered cells often skip the proliferation phase and enter the differentiation program. In other words, the cell proliferation, and also migration, are optimal at the intermediate adhesion strength (for a review, see^29,38^).

The parameters of adhesion of vascular endothelial cells in our study were unchanged or at most slightly elevated on the PP-coated dishes in comparison with unmodified ones (Fig. 2). This result is somewhat surprising because material surfaces enriched with amine groups usually improved the adhesion of endothelial cells. For example, silicon tubes modified with amine groups increased the adhesion strength of endothelial cells, which was manifested by their resistance to the detachment by fluid shear stress. Other chemical functional groups, namely carboxyl or peroxide groups, did not enhance the adhesion strength of endothelial cells, which was manifested by the detachment of more than 50 % of these cells under one hour-lasting fluid shear stress^39^. Similar results were obtained in an earlier study by Lee *et al*.^40^, performed on self-assembled monolayers (SAM) of alkylsilanes terminated with an epoxide, carboxyl (-COOH), amine (-NH_2_), and methyl (-CH_3_) groups. The strength of adhesion of human K100 erythroleukemia cells, determined by a spinning disc apparatus, was the highest on NH_2_-terminated alkylsilanes. This finding was attributed to non-specific cell adhesion such as electrostatic interactions between the cells and amine groups on the material surface, rather than to the specific receptor-mediated cell adhesion through proteins adsorbed on the material surface from the culture medium. When the alkylsilanes were functionalised with RGD oligopeptides, i.e., extracellular matrix-derived ligands involved in specific integrin-mediated cell adhesion, the strength of cell adhesion was nearly an order of magnitude lower than on NH_2_-terminated surfaces with non-specific cell adhesion^40^.

The observed increased resistance to trypsinization of myoblasts, fibroblasts, keratinocytes, and vascular smooth muscle cells on our NH_x_-terminated surfaces might also be explained by non-specific cell adhesion. The mechanism of action of trypsin is the cleavage of cell adhesion-mediating proteins and extracellular parts of integrin adhesion receptors, which cannot take place if the cells are directly bound to the material surface through NH_x_-groups. The non-specific adhesion of non-endothelial cells in our study and their direct adhesion to the PP surfaces through NH_x_-groups are also suggested by the resistance to trypsin in LF cells cultured in serum-free media, i.e., without cell adhesion-mediating proteins. This resistance was similar to the LF cells cultured in standard serum-supplemented medium (Fig. 2). This phenomenon suggested that the adhesion of LF cells was not caused only by binding the adsorbed cell adhesion-mediating proteins via cell adhesion receptors (e.g., integrins) but other factors, e.g., electrostatic interaction, played a significant role.

Another indirect sign of the non-specific cell adhesion is stagnating or even decreasing cell number from day 2 to day 7 in most of the studied cell types, which is most likely due to their low proliferation activity (Fig. 6). The non-specific, i.e., non-receptor-mediated cell adhesion, cannot ensure the transduction of proliferation signal to cells. This transduction is dependent on the cell adhesion receptors, associating the extracellular matrix and intracellular signalling and structural molecules (i.e., enzymes and cytoskeleton), and delivering biochemical and mechanical signals to cells (for a review see^25^).

The non-specific interactions between the NH_2_ groups on the material surface and cells are mediated by the glycocalyx, also known as the pericellular matrix, which is a complex of glycoproteins and glycolipids surrounding the cell membrane^40^. The glycocalyx can be retained, at least partly, on the cell surface even after trypsinization of cells before their seeding on the investigated materials. The glycocalyx endows the cell surface with a negative charge, which electrostatically interacts with positively charged -NH_2_ groups (in fact, -NH_3_^+^) groups on the material surface^40^.

The thickness, morphology, and biochemical composition of glycocalyx vary among cell types. For example, the glycocalyx of vascular endothelial cells degrades during vascular diseases, such as atherosclerosis and hypertension, and sepsis, particularly in its major components heparan sulfate (HS), hyaluronic acid (HA) and core proteins syndecans and glypicans. On the contrary, the glycocalyx on cancer cells is generally robust, with elevated HS, HA, and glypicans, and enhances the cell adhesion, migration, and growth^41,42^. Similar differences in glycocalyx could occur between endothelial cells and the other cell types investigated in the present study, and also among various endothelial cell lines. Differences in thickness and continuity of glycocalyx were found *in vitro* in three lineages of microvascular endothelial cells (MVEC), derived from various organs and species, namely in human dermal MVEC, murine cardiac MVEC and bovine MVEC derived from the corpus luteum^43^. The thickness of glycocalyx also vary in endothelial cells in various organs and along the vascular tree *in situ*, and even within a single blood vessel^44^.

Alteration of other extracellular matrix molecules, such as collagen and fibronectin, can also modulate the cell adhesion strength. In a study by Kemeny *et al*.^38^, glycated collagen, which occurs in blood vessels in diabetic patients, increased the adhesion strength (evaluated by a spinning disc apparatus) of porcine aortic endothelial cells (PAECs) *in vitro* in comparison with native collagen. It was attributed to binding of cells to glycated collagen through α_v_β_3_ integrin adhesion receptors instead of the α_2_β_1_ integrins, typically used by cells for binding to native collagen. In addition, PAECs showed higher adhesion strength when cultured in a medium with low glucose concentration (1 mM) or high glucose concentration (33 mM) than in a medium with normal glucose concentration (5.55 mM). This finding was explained by elevated VEGF release from cells in the low glucose medium, and by the elevated concentration of intracellular PKC in cells in high glucose medium^38^. However, an earlier study by Krantz *et al*.^45^ reported a decreased adhesion strength of bovine endothelial cells in cultures on glycated collagen and fibronectin^45^.

Another critical player in the adhesion of cells, particularly of endothelial cells, is tissue transglutaminase. It is a Ca^2+^-dependent GTP binding protein that crosslinks proteins *via* (epsilon)((gamma)-glutamyl)lysine bridges. This enzyme is located primarily in the cytosol, but it can be externalized and can play an essential role in the extracellular matrix organization, e.g., in fibronectin crosslinking^46^. Tissue transglutaminase co-localizes with beta_1_-integrin adhesion receptors^47^, and it has a positive influence on the phosphorylation of paxillin, which is vital for cell adhesion and migration^48^. Increased activity of tissue transglutaminase accompanied the increased resistance to trypsinization in murine sarcoma RIF-1 cells, HT29 human colonic carcinoma cells, and ECV304 human umbilical vein endothelial cells after photodynamic therapy using pyridinium zinc (II) phthalocyanine as sensitizer^37^.

In contrast, a reduced level of tissue transglutaminase in endothelial cells, obtained by their transfection with an antisense construct, decreased their spreading and adhesion strength, manifested by a lower resistance of these cells to trypsinization^46^. Besides, tissue transglutaminase is reduced in large arteries of hypertensive rats in comparison with control normotensive rats^49^. Also, in endothelial cells used in our study, the level of tissue transglutaminase might be reduced and might vary among the three cell lineages. However, this fact and all the causes of altered adhesion mentioned above need to be further investigated to clarify our observation.

In addition to non-specific cell adhesion mediated by electrostatic and other direct cell-material interactions, the composition of the cell culture media can markedly influence the cell attachment. Since this composition differed among the cell types used in our study, it could be responsible, at least partly, for the observed differences in cell adhesion and growth. The cell adhesion is affected by two main factors present in the culture media, particularly in their serum supplement, (i) molecules influencing the cell adhesion, and (ii) growth factors. The molecules affecting the cell adhesion include proteins mediating the cell adhesion (mainly vitronectin and fibronectin), and molecules non-adhesive for cells (albumin, high-density lipids). In 1987, a study by van Wachem *et al*.^50^ revealed that the adsorption of fibronectin from the serum of the culture medium to tissue culture polystyrene was maximum at a relatively low concentration of serum, i.e., 0.1 %, and then decreases with increasing serum concentration to its minimum at 10–20 %, i.e., the concentrations most frequently used for cell cultivation, including our study. At the same time, the 10–20 % concentration of serum promoted the maximum adsorption of the molecules non-adhesive for cells, namely albumin, and high-density lipids. Similar results were also obtained for poly(ethylene terephthalate) (PET, Dacron) and polytetrafluoroethylene (PTFE, Teflon), i.e., polymers used for fabrication of clinically used vascular prostheses^50^.

If the arguments discussed above are also valid for the amine PPs used in our study, all cells cultured in media with 10–20 % of serum adhered on surfaces covered mainly by cell non-adhesive molecules with a small amount of cell adhesion-mediating molecules. Only HSVEC and HUVEC cells, cultivated in a medium with 5 % serum, probably adhered to a substrate with a slightly higher amount of these molecules. Besides, their medium was enriched with growth factors. However, the cell behaviour, i.e., the kinetics of cell adhesion, proliferation, and low resistance to trypsin detachment was similar as in CPAE cells cultured in the same medium as C2C12, LF, and HaCaT cells, i.e., in a conventional medium with 10 % serum. Thus, the cell culture medium, that had different compositions according to varying requirements of the used cell types, was not the main factor responsible for the different behaviour of the cell types on the tested surfaces.

The similar resistance to the detachment by trypsin observed for the LF cells cultured in serum-supplemented and serum-free media (Fig. 2) supports the conclusion drawn in the previous paragraph. The factor explaining the difference in non-endothelial and endothelial cells interacting with amine PPs is most probably the non-specific direct adhesion of cells to the amine PPs through surface NH_x_-groups, that was more pronounced in non-endothelial than in endothelial cells.

Based on this conclusion, we expected a different pattern of focal adhesion plaques in endothelial and non-endothelial cells or on the PP and control surfaces. There was a difference among the cell types, but not as expected. One group of cells consisting of HUVEC, HaCaT, and LF cells, exhibied the perinuclear location of paxillin and peripheral localization of F-actin filaments already in a 2-hour-long experiment (Fig. 4). These cells attached quickly (Fig. 3) and spread even in the short incubation time except for the LF cells on the control dish (Fig. 4) that were roundish. However, on amine PP surfaces, the LF cells were highly spread, adhering by a large area (Fig. 4), and they were also able to attach exceptionally quickly to these surfaces (Fig. 3). These observations are in line with the high resistance of LF cells to trypsinization after cultivation in both serum-free and serum-supplemented media and suggest a high level of non-specific non-receptor mediated cell adhesion in LF cells. The distinctive pattern after two hours of incubation was observed in the second group of the cells, HSVEC and VSMC, that were not yet fully spread and were mostly round on both PP and control surfaces. Paxillin was located mainly on the cell periphery, together with F-actin, which was not organized in filaments. In longer (2 days) experiments, all cell types on both control and amine PP surfaces were well-spread and polygonal with similar localization of paxillin in the central part, and F-actin preferentially in the peripheral part of cells, where it was organized into filaments (Fig. 4).

Regarding the fact, that all cells in the 2-day-long experiment had perinuclear expression of paxillin and peripheral location of F-actin, we assume, that peripheral location of paxillin is bound to the transitional phase during the cell spreading. It has been reported than in the early phase of the cell spreading, immature paxillin-containing focal adhesion sites are formed mainly at the cell edges, while in a later stage of cell spreading, these sites become more mature and homogeneously distributed throughout the whole cell membrane contacting the cell adhesion substrate^29^.

Despite the interesting findings, we cannot explain the resistance to trypsin of non-endothelial cells by specific localization of focal adhesion plaques. We also tried to connect the circular or spread shape of cells to their ability to attach more rapidly on modified surfaces. However, VSMC cells were extremely quickly attached, and still, they are round in two hours of incubation. On the other hand, HaCaT cells growing on the control dish, do not attach quickly and they are already nicely spread in two hours. Thus, the reason for the different adhesion behaviour (i.e., preference of different mechanisms of cell adhesion) of endothelial and non-endothelial cells on the amine PPs remains to be further investigated.

## Conclusion

The central issue of this manuscript is the resistance of cells attached to amine plasma polymer surfaces to the trypsin treatment. We demonstrated the difference between endothelial and non-endothelial cells and also some differences among tested cell types. The increased resistance to trypsin treatment observed for the non-endothelial cell lines was accompanied by an increased rate of cellular attachment, even though spontaneous migration was comparable to the control, i.e., the standard cultivation surface. The increased cell adhesion was confirmed for LF cells also by an independent technique, single-cell force spectroscopy. This method, as well as the cell attachment rate, proved the difference among the PPs with different amounts of amine groups, but other investigated techniques could not reveal the differences in the cell behaviour on different amine PPs.

Based on all the results, the increased resistance to trypsinization of myoblasts, fibroblasts, keratinocytes, and vascular smooth muscle cells on our amine plasma polymer surfaces can be explained most probably by a non-specific cell adhesion such as electrostatic interaction between the cells and amine groups on the material surface. The differences among the particular cell types are most plausibly caused by different compositions of their glycocalyx and expression of transglutaminases.

The possible use of our finding is not only limited to the engineering of active tissues, but amine-rich layers can also be used for “glueing” semi-adhesive or even suspension cells to the surface in different biological applications, e.g., immunocytochemistry. Importantly, the plasma-chemical way of preparing such “amino-glue” is much less time consuming than the chemical preparations, e.g., preparation of polydopamine-based coating, and it is compatible with dry patterning techniques. To sum up, amine-rich surfaces can modulate adhesion and proliferation of cells, which could be beneficial for numerous applications.

## Methods

### Preparation of Plasma Polymers

The amine PPs films were deposited in radio frequency (RF) discharge with capacitive coupling from cyclopropylamine (CPA) vapours mixed with Ar. The procedure and the film analyses have been already reported in the previous publications^23,25^. For the current studies, the four types of amine PPs films were selected. They were prepared at the pressure 50 Pa and with the CPA:Ar flow rate ratio of 2:28. The film deposition conditions differed in the RF power invested into the plasma-chemical reaction. In three cases, the RF (13.56 MHz) discharge was operated in the pulsed mode using the duty cycle 33 % and the repetition frequency of 500 Hz. The on-time RF power was 30, 50 and 100 W. The fourth sample was prepared in continuous wave discharge (duty cycle 100 %) at the RF power of 150 W. To simplify the space of the deposition parameters, the films are identified by the average RF power (${P}_{\text{av}}$) calculated as the on-time RF power multiplied by the duty cycle. Thus, the four above listed samples have ${P}_{\text{av}}$ of 10, 17, 33 and 150 W.

### Characterization of Plasma Polymers

The CPA plasma polymers were characterized previously for their chemical composition and chemical bonds by X-ray photoelectron spectroscopy (XPS)^25^. The analyses were carried out with a non-monochromatic Omicron X-ray source (DAR400, output power 270 W) and an electron spectrometer (EA125) attached to a custom-built ultra-high vacuum chamber. The elemental composition was quantified by XPS MultiQuant software from the XPS spectra taken at the pass energy of 25 eV and electron take-off angle 50°. The maximum lateral dimension of the analysed area was 1.5 mm. To determine the percentages of different carbon and nitrogen bonds, the fitting of XPS C1s and N1s signals was performed in the CasaXPS software version 2.3.17 after subtraction of the Shirley background by employing Gaussian–Lorentzian (G-L) peaks with the fixed G-L percentage 30 % and the full width at a half maximum set to 1.85 ± 0.05 eV. The C1s signal was fitted by a sum of three components corresponding to hydrocarbons (**C**H_x_ = 285.0 eV), carbon bonded to nitrogen or oxygen (**C**-N/**C**-O/**C**≡N ~ 286.3 ± 0.1 eV) and carbon double bonded to oxygen (N-**C**=O/-**C**=O ~288 ± 0.1 eV). The N1s signal was fitted by a sum of three components corresponding to nitride/imine (**N**=C ~398.2 ± 0.2 eV), the amine group (**N**H_x=1,2_ ~ 399.1 ± 0.1 eV) and amide/nitrile group (**N**-C=O/C≡**N** ~ 400.2 ± 0.1 eV).

The film stability in water was also studied previously^25^ by measuring the change of film thickness after 216 hours of immersion in the de-ionized water at room temperature (~25 °C). The relative thickness change for the films deposited on silicon substrate was determined from ellipsometric data obtained before and after the water immersion using Jobin Yvon UVISEL ellipsometer in the spectral region of 1.5–6.5 eV at angle of incidence of 65°.

Topography of coated and uncoated dishes was measured using Dimension Icon (Bruker) atomic force microscope. Images of 30 µm × 30 µm areas were acquired with the step of approx. 6 nm in the tapping mode with RTESPA-150 probes (Bruker), in at least 5 locations (total) of 3 different dishes for each sample type. Mean square roughness was calculated according to ISO 25178 and correlation length was obtained by fitting the fast-axis autocorrelation function using the Gaussian model after the second-order scan line background subtraction in Gwyddion^51^.The water contact angle (WCA) was measured on coated dishes by a SEE System 7.0 sessile drop technique enabling the observation of solid-liquid meniscus using a CCD camera. During the measurement, the shape of the water droplet changed after touching of sample surface; namely, the WCA decreased gradually. Therefore, the drop snapshots were captured with ~0.2 s delay after the droplet touched the surface to ensure correct comparison. The WCA values were calculated based on the tree-point interpolation of the drop height and width from the images.

### Cultivation of Cells

Cultivation of mouse myoblasts (C2C12), human skin fibroblasts (LF), human keratinocytes (HaCaT), vascular smooth muscle cells (VSMC), and bovine endothelial cells (CPAE) was carried out in constant incubator conditions (37 °C, air atmosphere with 5 % CO_2_, and 95 % humidity) in DMEM (Dulbecco’s modified Eagle’s medium, high glucose, Gibco, Thermo Fisher Scientific, Waltham, Massachusetts, USA) with the addition of 10 % FBS (fetal bovine serum, Gibco, Thermo Fisher Scientific), 1.2 mM L-glutamine (Gibco, Thermo Fisher Scientific) and 100 U/ml Penicillin/Streptomycin (HyClone, Thermo Fisher Scientific); only the VSMC cells had media enriched with 20 % FBS. The cells for following experiments were used in the range of 10 passages, appropriately for particular cell type. The VSMC cells are originally primary culture, but with extended cultivation till 25^th^ passage. For following experiments we used cell passages 15–25. Cultivation of human endothelial cell lines HUVEC and HSVEC was carried out in the same incubator in Endothelial Cell Growth Medium 2 with the following supplements: 5 % FCS, human Epidermal Growth Factor, Vascular Endothelial Growth Factor, basic Fibroblast Growth Factor, R3 Insulin-like Growth Factor-1, Ascorbic acid, Hydrocortisone, Heparin (PromoCell, Heidelberg, Germany) and 100 U/ml Penicillin/Streptomycin (HyClone, Thermo Fisher Scientific). For those endothelial cells, only the first 5 passages were used for experiments as they are primary cultures.

Cells in the stock passage and control cells were cultured in a standard laboratory tissue culture dishes (40 mm × 10 mm, volume 2 ml, TPP, Merck, Kenilworth, New Jersey, United States). The same Petri dishes coated by amine PPs layers were used for the experiments. The cells were seeded in concentration of 1 × 10^5^ cells per dish.

During passaging, phosphate buffered saline (PBS; pH 7.4) was used to rinse all types of cells, and they were then enzymatically released from the surface by 1 × trypsin-EDTA (ethylene-diamine tetraacetic acid, Biotech, LM-T1706/100, Onsala, Sweden). Inversion microscope CKX 41 (Olympus, Tokyo, Japan) was used for visual inspection of cells during passage and experiments.

### Trypsinization

To measure the level of cell resistance to trypsinization, we used treatment with 1 x trypsin-EDTA and evaluated the detachment of cells from the plasma coated versus control Petri dishes. The dishes were seeded with 1 × 10^5^ cells cultivated at the standard conditions described above. A further experiment with the cultivation without serum was performed for the LF cell line. For the serum-free tests, we substituted DMEM medium by DMEM-F12 medium (Gibco) and FBS by ITS supplement (containing insulin, transferrin, selenium, Gibco, 100-fold diluted).

After 24 hours of cultivation (16 hours in the case of serum-free tests), the surface was rinsed with PBS, and 1x trypsin-EDTA was applied. Samples were then incubated at 37 °C, and the pictures were taken at time 0, 10, 20, and 30 minutes by the microscope IX51 using a Camedia camera (both Olympus).

The results were evaluated using the 1.51w Fiji (Wayne Rasband, NIH, USA). In each condition, four field shots focused on the surface were taken, which were automatically crafted with intensity, saturation and colour clarity. Subsequently, the binarization of the acquired mask and its optimization were performed using image analysis tools. The results were obtained by automatic counting of particles larger than 600 pixels, and in graphs given as the average number of sessile cells per field of view. For each film and cell type 4 independent measurements were done. Each time dependence was first normalised independently by dividing by the value for time t = 0 s. The variance of this value was taken into account in the variances of plotted values at other times using the standard error propagation rule.

### ATP assay

The method of determining the relative amount of ATP in cell lysate is based on assessment of the level of chemiluminescence emitted by luciferase, which cleaves luciferin as long as there is any ATP left. Cells were cultured for 2, 4 and 7 days on treated and control Petri dishes and rinsed with PBS. Then, 200 μl of the Somatic cell ATP release reagent (Sigma Aldrich, Merck KGaA, Darmstadt, Germany) was added to each plate and incubated in RT for 10 minutes on shaker. The lysate was mixed in a 1:1 ratio with a solution containing luciferase and luciferin mixture (BioThema, Handen, Sweden). In the shortest possible time, chemiluminescence was measured on the LMT 01 luminometer (Immunotech, Monrovia, USA) using the Microwin 2000 program (Microtel, USA). For each film and cell type combination, at least 6 measurements were done. The relative value to control was calculated independently for each using first-order bias-corrected ratio estimator.

### Time-lapse microscopy

Cells cultivated as mentioned above and seeded in particular 2 ml dish were immediately inserted in the cultivation chamber of Live Cell Imaging system (Olympus IX83). The time-lapse images were captured for 20 hours in every 5 minutes. The sequence of tiffs was converted to the tiff video using Fiji^52^. This sequence was uploaded to the Itrack4you software^53^, and the pre-processing was performed. Then 40 cells were manually assigned in each sequence (3 sequences for every combination of cell and surface type), and the tracking was performed automatically in reverse mode - from the end of the video to its beginning. Cells present in fewer than three pictures were omitted from the evaluation of average cell velocity. The average velocity was estimated as the arithmetic mean (with corresponding simple variance estimate) of all distances that any tracked cell moved between two frames, divided by the frame time step (5 minutes). It was calculated from at least 13 data points.

The same sequence of images was also used for assessing the rate of attachment of freshly seeded cells and for the next 75 minutes. In each of 15 consecutive images, cells attached to surface were counted. The total number of cells was also assessed.

Cell morphology was evaluated using images taken ten hours after seeding, before cells began to exhibit signs of apoptosis. For each cell and dish type 20 cells were measured with Fiji^52^. Cell length $L$ was measured as the maximum bounding dimension (maximum Feret’s diameter), and cell width $W$ as the Feret’s diameter in the perpendicular direction. Their mean values and standard deviations were estimated using standard elementary estimators. Aspect ratio $r$ for each dish and cell type was obtained by linear orthogonal regression with the model $L={rW}$, assuming $L$ and $W$ have independent errors and equal variances.

### Fluorescence imaging by confocal microscopy

The coverslips (Marienfeld-Superior, Germany) coated with the plasma polymer deposited at ${P}_{\text{av}}=33$ W helped to visualize the cell-surface interactions by the confocal microscopy. Cells were cultivated in dishes with the PP-coated and control coverslips for 2 hours or two days. Then, we washed the coverslips with PBS and fixed the cells in 4 % paraformaldehyde (15´/RT). We laid the coverslips on parafilm (M, Bemis Flexible packaging) with cells facing down on a drop of blocking buffer (5 % bovine serum albumin in PBS) for 30 minutes in a wet chamber and at room temperature. We washed the cells 3-times by adding an excess amount of PBS and incubated them for 2 hours in a wet chamber in primary antibody anti-paxillin (AB3794, Millipore, Merck, 1:200 in the incubation buffer - 5 % BSA and 0.1 % TRITON in 2x PBS). After washing 3-times with PBS, the secondary antibody was applied for one hour (anti-rabbit-Alexa 488, Invitrogen by Thermo Fisher Scientific, 1:500 in the incubation buffer) together with the phalloidin-TRITC to visualize F-actin (P1951, Sigma Aldrich, 1:200 in the same incubation buffer). After washing, the coverslips were mounted on the carrier glass with fluorescence mounting medium (DAKO, Glostrup, Denmark). The pictures were taken with the scanning confocal microscope Leica SP8 (Leica Microsystems GmbH, Wetzlar, Germany).

### Single-cell force spectroscopy

Single-cell force spectroscopy (SCFS) of LF cells was performed using a JPK CellHesion 200 (z-range 0–100 μm), mounted on inverted optical microscope Olympus IX-81S1F-3 and equipped with temperature-controlled PetriDishHeater (JPK), keeping medium temperature at 37 °C. Arrow-TL2 tipless cantilevers (NanoWorld) with length 500 µm and nominal spring constant 0.03 N/m (calibrated individually by the thermal noise method^54^ were utilized. They were functionalised by gelatine type A (Sigma-Aldrich) for cell immobilization after cleaning in piranha (1:3) solution.

For this experiment were LF cells propagated on 100 mm Petri dish in MEF medium, which consists of Knockout Dulbecco’s modified Eagle’s medium (KO-DMEM; Gibco), 10 % heat-inactivated FBS (Invitrogen), 1 % L-glutamine (Gibco), 1 % non-essential amino acids (PAA), 1 % penicillin-streptomycin (PAA), and 0.1 mM β-mercaptoethanol (Sigma), until passage 2. The cells were washed with PBS and trypsinized (trypsin-EDTA, Invitrogen) for 2 min and collected into centrifuge tube. The cell suspension was spun by 200 g for 4 min and resuspended to the concentration 10^5^ cells/ml in 1 ml of KO-DMEM medium, then used for SCFS experiments.

A single cell was picked up from the uncoated substrate region and captured in the cantilever tip area (contact time 120 s). For the measurement, the cantilever was lowered from height approximately 80–95 µm above the surface with the constant speed of 5 µ/s until the cell contacted the surface. During subsequent 20 s of contact the force was kept constant at 2 nN. The contact time had been chosen, based on preliminary measurements, as optimal for substantial adhesion response, but not yet too large to damage the cells quickly^55,56^. Finally, the cantilever was retracted with the same speed to the original height.

Comparative measurements to uncoated Petri dish (area covered by a cover slip during deposition) were performed by alternating between coated and uncoated surfaces after 3 force curve acquisitions. At least 70 curves in total on two different dishes were measured for each coating type. Baseline, offset and slope adjustments were carried out in JPK Data Processing (version spm-6.0.31); parameters F_D_, W_D_ and number of t-steps^56,57^ were evaluated by a numpy script. Exponential time dependences of the parameters^58,59^ were observed, each time dependence for the uncoated surface was thus fitted by an exponential. The coated/uncoated ratio was then calculated by dividing the value for coated surface by the value of the corresponding uncoated exponential for the same time. The bias of this ratio estimator was first-order corrected using the estimated geometric standard deviation of uncoated values with respect to the exponential and assuming they have a log-normal distribution.

## Supplementary information

Supplementary Information.

## Data Availability

The datasets generated during and/or analysed during the current study are available from the corresponding author on reasonable request.
