# Supplementary material for: Cell type specific adhesion to surfaces functionalised by amine plasma polymers
Source: Sci Rep. 2020 Jun 9;10:9357. doi: 10.1038/s41598-020-65889-y (PMC7283471; doi:10.1038/s41598-020-65889-y)
Supplement: Supplementary file 1 — Supplementary Information. [file 41598_2020_65889_MOESM1_ESM.pdf]

Supporting Information

Cell type specific adhesion to surfaces functionalized by amine plasma polymers

P. Černochová[1,2], L. Blahová[2], J. Medalová[1,2], D. Nečas[2,3], M. Michlíček[2,4], P. Kaushik[2,4], J. Přibyl[5], J. Bartošíková[1], A. Manakhov[2,6], L. Bačáková[7], L. Zajíčková[2,3,4]

[1] Department of Experimental Biology, Faculty of Science, Masaryk University, Kamenice 5, Brno 625 00, Czech Republic

[2] RG Plasma Technologies, Central European Institute of Technology – CEITEC, Masaryk University, Kamenice 5, Brno 625 00, Czech Republic

[3] Central European Institute of Technology – CEITEC, Brno University of Technology, Purkyňova 123, Brno 612 00, Czech Republic

[4] Department of Physical Electronics, Faculty of Science, Masaryk University, Kotlářská 2, Brno 611 37, Czech Republic

[5] Core Facility Nanobiotechnology, Central European Institute of Technology – CEITEC, Masaryk University, Kamenice 5, Brno 625 00, Czech Republic

[6] Scientific Institute of Clinical and Experimental Lymphology– Branch of the ICG SB RAS, 2 Timakova str., 630060 Novosibirsk, Russian Federation

[7] Institute of Physiology of the Czech Academy of Sciences, Vídeňská 1083, Prague 142 20, Czech Republic

1. Atomic force microscopy of cultivation dishes coated by plasma polymers

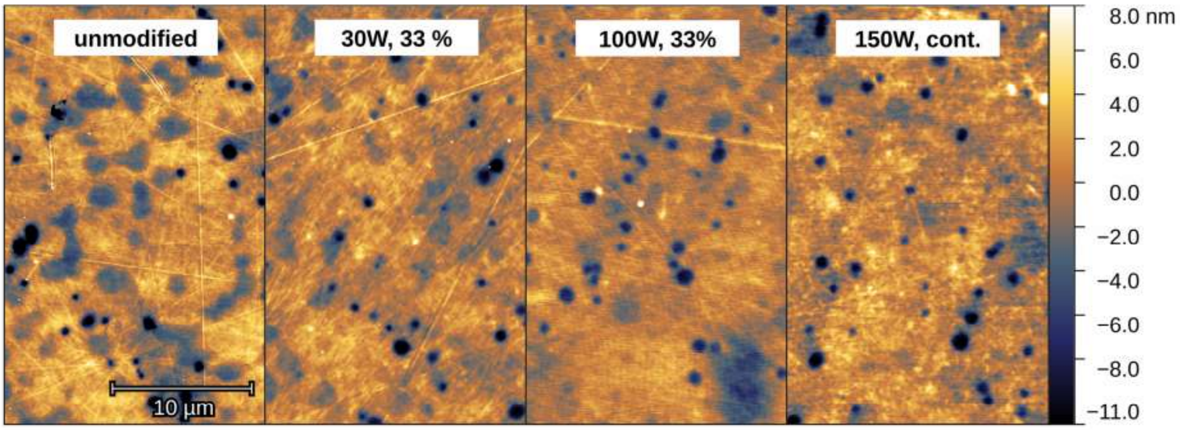

Fig. S1 Character of surface topography of uncoated dishes and dishes with deposited PPs, as measured by AFM. All images have the same height scale, indicated on the right.

Table S1 Surface roughness parameters of uncoated dishes and dishes with deposited PPs, as measured by AFM. The mean square roughness characterises height of roughness features whereas the correlation length characterises their lateral dimensions.

Sample	Mean square roughness [nm]	Correlation length [µm]
uncoated dish	5.7 ± 0.9	1.9 ± 0.5
30 W, pulsed 33 %	3.4 ± 0.8	1.5 ± 0.4
100 W, pulsed 33 %	5.4 ± 1.2	1.8 ± 0.3
150 W, continuous	4.4 ± 0.8	1.6 ± 0.4

2. Cells imaged by inverted optical microscope

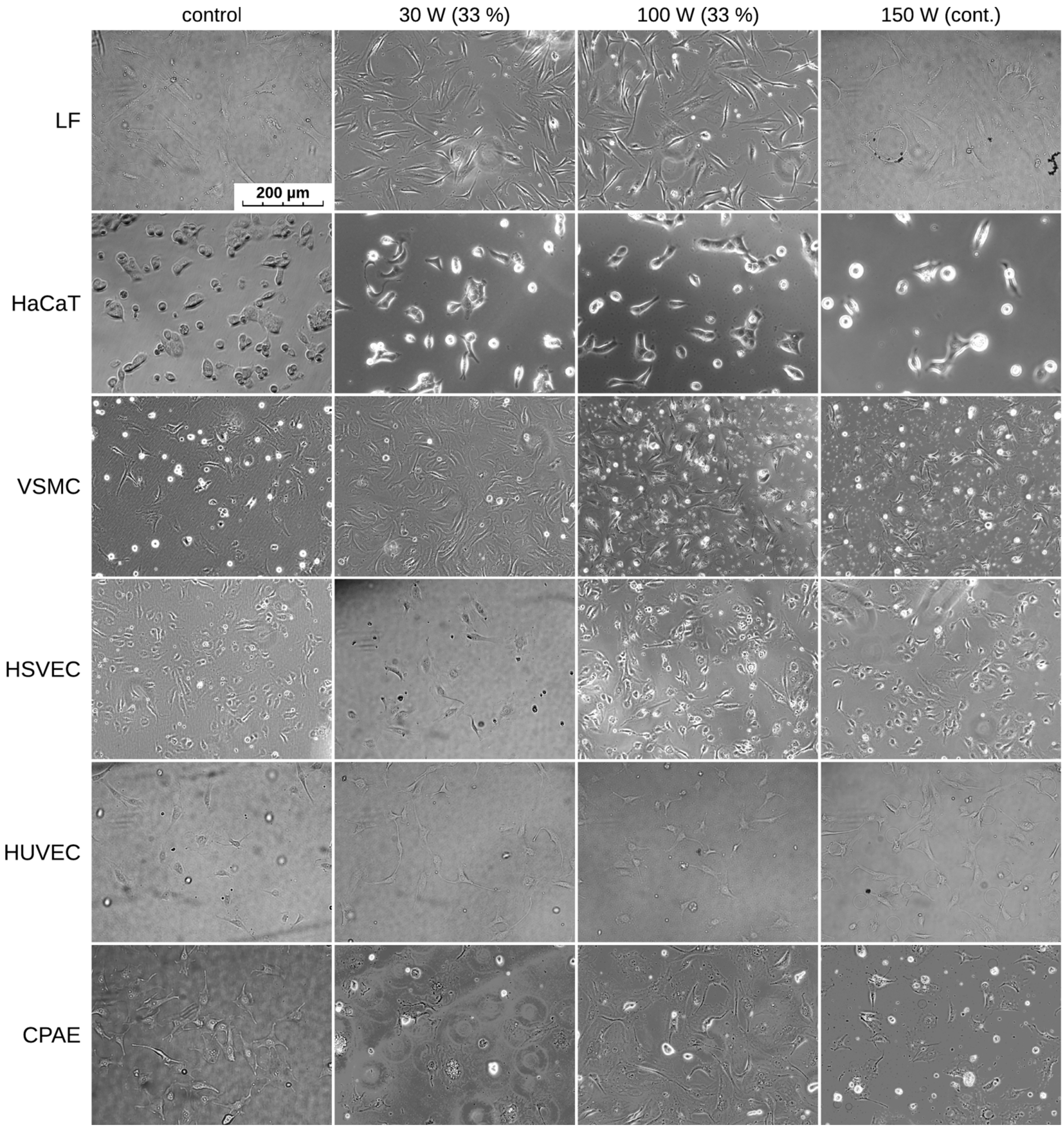

Fig. S2 Typical optical images of the cells incubated ten hours in the chamber of time-lapse microscope. The images were evaluated for the cell length, width and aspect ratio summarized in Fig. S3.

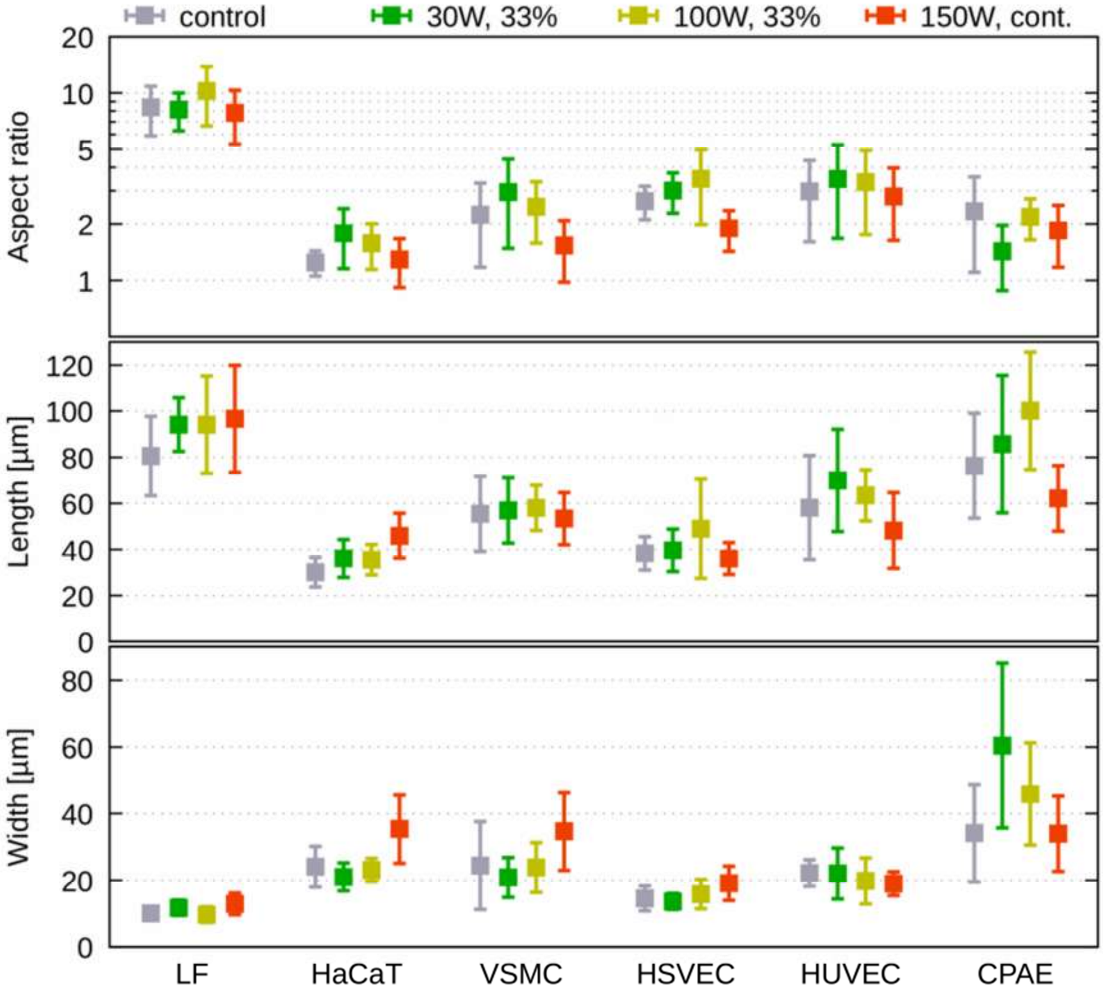

Fig. S3 Cell length, width and aspect ratio determined by inverted optical imaging. The results are expressed as a mean with corrected sample standard deviation.

3. Fluorescence imaging of cell-surface interaction

We visualized (i) the focal adhesion plaques with immunofluorescence staining of paxillin, a protein closely associated with integrin adhesion receptors, and (ii) the actin cytoskeleton with staining of filamentous actin (F-actin) using phalloidin conjugated with a fluorescence marker TRITC. The confocal images for all the cells, i.e. LF, HaCaT, HUVEC, CPAE, HSVEC and VSMC, on control (uncoated) and amine plasma polymer surfaces are in Fig. S4. In 2-hour-long experiments, the cells displayed two different staining patterns (i) LF, HaCaT, HUVEC, and CPAE cells were able to spread nicely even in this short time, with the only exception of LF cells growing on the control surface, (ii) HSVEC and VSMC were round on both the PP and control surfaces.

Human skin LF fibroblasts on the control polystyrene (PS) surfaces were round, non-spread and without assembled focal adhesion plaques and filamentous actin cytoskeleton. At the same time, the LF cells the PP surface were fully spread with well-developed F-actin cytoskeleton already 2 hours after seeding, although the paxillin-containing focal adhesion plaques were less apparent and were probably masked by a high autofluorescence of the PP samples (Fig. S4). This observation is in accordance with a markedly higher fraction of attached cells (Fig. 3 in the manuscript), and also with a higher resistance to trypsinization of LF cells on PPs than on control PS (Fig. 2 in the manuscript). However, on day 2 after seeding, the LF fibroblasts were equally spread with well-developed F-actin cytoskeleton on both types of surfaces, although the paxillin-containing focal adhesion plaques were still less apparent on PP surfaces. Generally, in all cell types and on all tested surfaces, paxillin-containing focal adhesion plaques were very small and of a fine granular appearance.

Human HaCaT keratinocytes appeared to be equally spread on both control PS surfaces and PP surfaces 2 hours after seeding, although the focal adhesion plaques and actin cytoskeleton were masked by a high autofluorescence of PP surfaces. On day 2 after seeding, the HaCaT cells on both types of surfaces started to form islands, which are typical for keratinocytes. This is in line with a relatively good proliferation of HaCaT cells from day 2 to day 7 after seeding, which on days 4 and 7 almost reached the level of proliferation on control PS surface, at least on the PP deposited at 150 W (Fig. 6 in the manuscript), i.e. with the lowest amount of amine groups and thus probably with the lowest level of non-specific direct cell-material adhesion.

Endothelial HUVEC cells 2 hours after seeding were slightly better spread on the control PS than on the PP surface. On PS, the F-actin was homogeneously distributed throughout the entire cells, while on PP surfaces, it was located mainly at the cell periphery. On day 2 after seeding, the cells were equally spread on both types of tested surfaces, and showed similar distribution of focal adhesion plaques and actin cytoskeleton. This can explain the trypsinization behavior of these cells, which was almost similar on both PP and PS surfaces (Fig. 2 in the manuscript).

Endothelial CPAE cells displayed a similar picture of cell spreading and distribution of paxillin-containing focal adhesion plaques and F-actin cytoskeleton as HUVEC cells. The cells were well-spread on both control PS and PP surfaces, although the cell spreading area was larger on PS surfaces, which was apparent mainly on day 2. Paxillin was visible predominantly in the central part of cells, while actin was located mainly at the cell periphery, which was well-apparent particularly on control PS samples, i.e. samples without autofluorescence.

Endothelial HSVEC cells were round and non-spread 2 hour after seeding on both control PS and investigated PP surfaces. On day 2 after seeding, the HSVEC were spread on both types of surfaces, although they seemed to be better spread and with better-developed focal adhesion plaques and actin cytoskeleton on control PS than on PP surfaces. Similar picture was also observed in VSMCs.

Vascular smooth muscle cells (VSMC) were round and non-spread 2 hour after seeding on both PP and control PS and investigated PP surfaces. On day 2, the cell on both surfaces were spread and polygonal, but the adhesion area was larger and the focal adhesion plaques and actin cytoskeleton were better formed in cells on control PS surface than on PP.

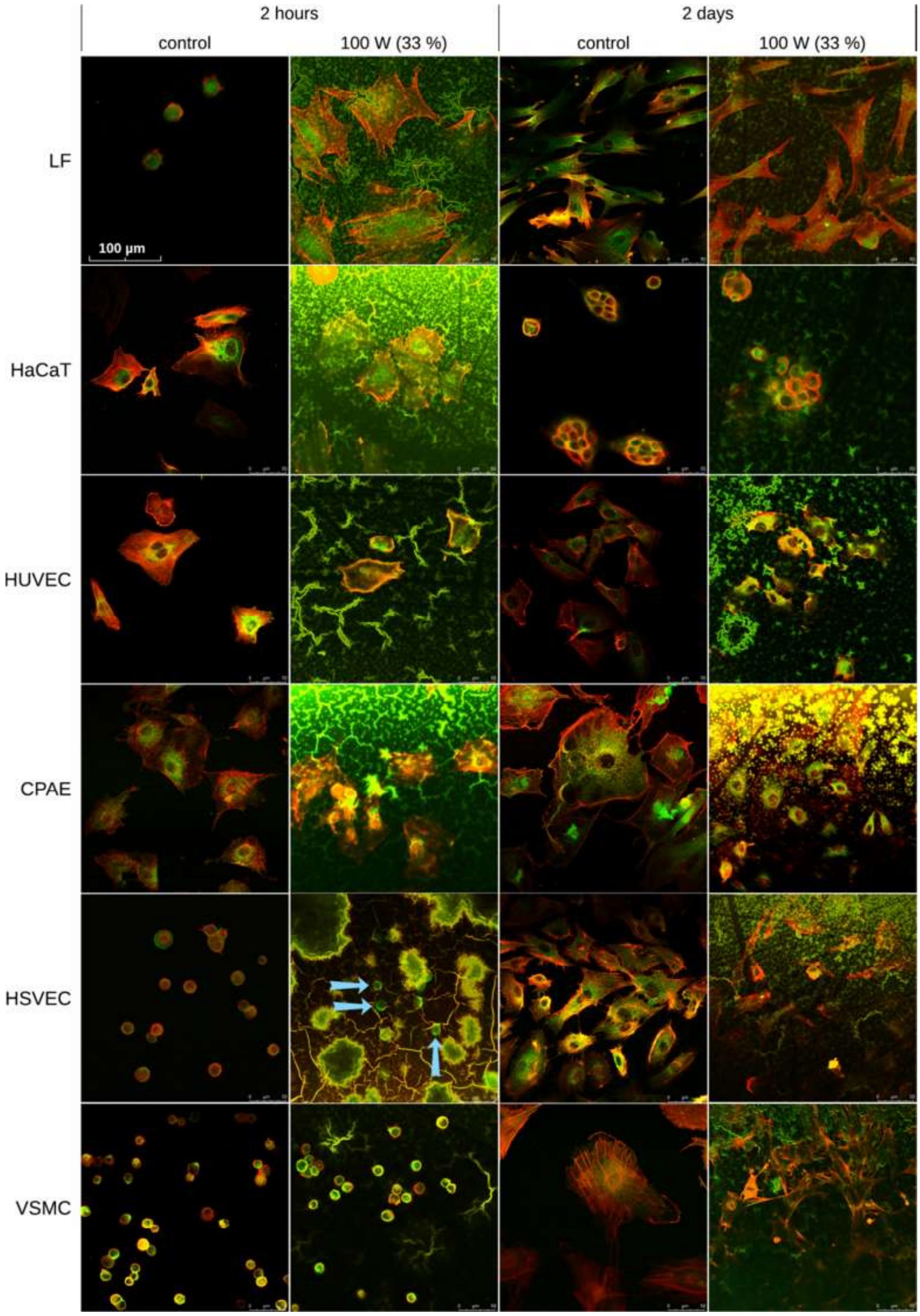

Fig. S4 Visualization of cell-surface interaction by immunofluorescence staining of focal adhesion plaques (paxilin, green fluorescence) and fluorescence staining of actin cytoskeleton by TRITC-conjugated phalloidin (F-actin, red fluorescence). Blue arrows point to the cells.
